# Distinct characteristics of Tregs of newborns of healthy and allergic mothers

**DOI:** 10.1371/journal.pone.0207998

**Published:** 2018-11-26

**Authors:** Viktor Černý, Jiří Hrdý, Olga Novotná, Petra Petrásková, Kristýna Boráková, Libuše Kolářová, Ludmila Prokešová

**Affiliations:** 1 Institute of Immunology and Microbiology, First Faculty of Medicine, Charles University and General University Hospital in Prague, Prague, Czech Republic; 2 Institute for the Care of Mother and Child, Prague, Czech Republic; Wayne State University School of Medicine, UNITED STATES

## Abstract

Allergic diseases represent a major issue in clinical and experimental immunology due to their high and increasing incidence worldwide. Allergy status of the mother remains the best predictor of an individual’s increased risk of allergy development. Dysregulation of the balance between different branches of immune response, chiefly excessive polarization towards Th2, is the underlying cause of allergic diseases. Regulatory T cells (Tregs) play a pivotal role in the timely establishment of physiological immune polarization and are crucial for control of allergy. In our study we used flow cytometry to assess Tregs in cord blood of newborns of healthy (n = 121) and allergic (n = 108) mothers. We observed a higher percentage of Tregs (CD4^+^CD25^+^CD127^low^FoxP3^+^) in cord blood of children of allergic mothers. However, the percentage of cells expressing extracellular (PD-1, CTLA-4, GITR) and intracellular (IL-10, TGF-β) markers of function was lower (significantly for PD-1 and IL-10) within Tregs of these children. Furthermore, Helios^-^ induced Tregs in the cord blood of children of allergic mothers were decreased. These results were supported by a decrease in plasma levels of IL-10 and TGF-β in cord blood of newborns of allergic mothers, implying lower tolerogenic capacity on the systemic level. Taken together, these findings reflect deficient function of Tregs in the group with higher risk of allergy development. This may be caused by a lower maturation status of the immune system, specifically Tregs, at birth. Such immaturity may represent an important mechanism involved in the increased risk of allergy in children of allergic mothers.

## Introduction

Allergic diseases belong to the most common and important medical conditions. Despite intensive research, the early events leading to the development of allergy in predisposed infants remain to be conclusively elucidated. The hygiene hypothesis is a major theory, postulating that lower exposure to microbes typical for the more developed countries may delay the development of the immune system and alter the balance among immune response branches (e.g. Th1, Th2, Treg, Th17), facilitating allergy. Prenatally, T helper type 2 (Th2) response is favoured to prevent undesirable reactivity towards maternal antigenic determinants foreign to the foetus [1]. Beginning after birth, a new physiological balance needs to be established upon contact with external environment, chiefly upon exposure to microbial stimuli. Persistence of the Th2 bias predisposes towards allergy development; Th1 and Th17 responses play important roles in anti-infectious immunity, but under certain conditions can lead to the development of autoimmune diseases. Regulatory T cells (Tregs) are the master T lymphocyte population overseeing this fine tuning and controlling potential development of pathological reactions including allergy-associated Th2 responses [2].

Human Tregs represent a population of CD4^+^ T cells characterised by a typical set of cell surface markers (CD4^+^CD25^+^CD127^low^) and intracellular markers (FoxP3^+^, Helios^+^). FoxP3 can be considered as a master transcription factor of Tregs responsible for their regulatory functions, and MFI of FoxP3 has been shown to correlate with Treg suppressive function [3]. Another hallmark of Tregs is a high dependence on IL-2. Tregs play an indispensable role in maintaining immunological reactions within physiological proportions, as evidenced by the severe autoimmune phenotype seen in FoxP3 deficient patients [4]. They are also essential for controlling allergy [2] and form the basis of allergen-specific immunotherapy [5]. Tregs exert their immunosuppressive function in a number of biologically significant ways, both through direct cell to cell contact (e.g. CTLA-4, LAG-3, PD-1, FasL) and remotely through the secretion of immunoregulatory cytokines (IL-10, TGF-β, IL-35) [6]. Measurement of these and other related markers is therefore routinely used to indirectly assess Treg´s suppressive capabilities and functional status.

Numerous Treg subpopulations differing in the details of their localization and function as well as expression of markers have been described, making proper identification strategies vital for data reproducibility [7]. Conventional CD4^+^CD25^+^CD127^low^FoxP3^+^ Tregs can be divided into two broad groups with distinct origin and function: natural Tregs (nTregs) arise in thymus, possess TCR specificity mainly towards autoantigens and have been described to express the Ikaros family transcription factor Helios [8–10] and occasionally also neuropilin-1 [11], while Helios^-^ induced Tregs (iTregs) take on Treg phenotype upon interaction with environmental or self- antigens within the context of an immunosuppressive cytokine milieu (mainly TGF-β) on the periphery [12].

Many groups have so far tried to identify early prognostic markers which could be used to predict increased risk of allergy development, however with little conclusiveness. Maternal allergy remains the strongest and most reliable universally accepted established risk factor [13]. Among parameters studied in the cord blood were immunoglobulin (Ig)E levels [14,15], Th1 and Th2 cytokine proportions in plasma [16,17] as well as responsiveness of cord blood cells to various modes of stimulation [13,18,19]. Several groups including our own have reported correlation between proportional and functional characteristics of Tregs in cord blood and allergy status of the mother [7] or early allergy disorders in infants [20,21]. Our own observations revealed a lower presence of functional markers (IL-10, TGF-β, MFI of FoxP3) in Tregs from cord blood of children of allergic mothers as well as increased size of the population, possibly due to a compensatory upregulation caused by the dysfunctional nature of these cells [7]. We postulated that lower overall perinatal maturity of the immune system in children of allergic mothers may be the underlying cause of this. To further elucidate the relationships among functional phenotype of Tregs, their maturation status and risk of allergy development, we analysed Tregs and compared their populations as well as chosen surface (CTLA-4, PD-1, GITR) and intracellular (IL-10, TGF-β) markers of Tregs in cord blood of new-borns of allergic mothers (children with a relatively high risk of allergy development) and of healthy mothers (low-risk children). Importantly, we also compared the proportions of Helios^+^ Tregs (putative nTregs) and Helios^-^ Tregs (putative iTregs), as we postulated that since iTreg arise mostly due to postnatal exposure to harmless exogenous antigens, their decreased number may reflect a lower overall maturation status of Tregs even on the level of cord blood.

## Materials and methods

### Subjects and sample collection

Healthy (n = 121) and allergic (n = 108) mothers with physiological pregnancies who delivered children vaginally at full term in the Institute for the Care of the Mother and Child in Prague, Czech Republic, were included for the study. There was no difference in pregnancy length between the two groups. Allergy status of the mother was determined based on clinical manifestation of allergy persisting for at least 24 months; allergy against respiratory and/or food allergens manifested by various individual combinations of symptoms (e.g. hay fever, conjunctivitis, eczema, bronchitis, asthma etc.), monitoring by an allergist, positive skin prick tests or positive specific IgE and anti-allergic treatment before pregnancy. The study was approved by the Ethical Committee of the Institute for the Care of Mother and Child (Prague, Czech Republic) and was carried out with a signed written informed consent of the mothers.

Cord blood (CB) samples (approx. 5 ml) were collected into sterile heparinized tubes immediately after birth via umbilical vein punction, as described previously [17]. CB plasma was obtained for cytokine and IgE detection. Mononuclear cell fraction was obtained from whole cord blood by density gradient centrifugation (Histopaque-1077; Sigma-Aldrich, St. Louis, MO, USA) for culture assays.

### Flow cytometry

Whole blood samples were prepared and stained for flow cytometry as described in our previous studies [7]. Briefly, samples of whole blood were stained with the following antibodies against Treg surface markers: CD4 fluorescein isothiocyanate (FITC; clone RPA-T4; Becton Dickinson, Franklin Lakes, NJ, USA), CD25 peridinin chlorophyll-cyanin 5.5 (PerCP-Cy5.5; clone MEM-181; Exbio pls., Vestec, Czech Republic), and CD127 phycoerythrin-cyanin 7 (PE-Cy7; clone A019D5; BioLegend, San Diego, CA, USA). Staining and sample preparation were performed according to manufacturer’s instructions using human regulatory T cell whole blood staining kit (eBioscience, San Diego, CA, USA). After fixation and permeabilization, the samples were stained with antibodies against Treg intracellular markers: FoxP3 phycoerythrin (PE; clone PCH101; Thermo Fisher Scientific, Waltham, MA, USA) and Helios allophycocyanin (APC; clone 22F6; BioLegend). In some experiments, non-stimulated whole blood samples were stained for the following surface markers associated with Treg function: CTLA-4 APC (clone L3D10), PD-1 allophycocyanin- cyanin 7 (APC-Cy7; clone EH12.2H7), GITR PE (clone 621), all from BioLegend. Non-stimulated whole blood samples treated with BD GolgiPlug (Becton Dickinson) for 6 hours were permeabilized after surface staining for Tregs and stained for intracellular expression of regulatory cytokines IL-10 PE (clone JES3-19F1) and TGF-β PerCP-Cy5.5 (clone BG/hLAP), both from BioLegend. Gating strategy used for estimation of Treg was described in greater detail previously [22]. Briefly, lymphocyte gate was set based on forward-scatter (FCS) and side-scatter (SSC) characteristics with doublets exclusion (FCS-A × FCS-H; S1A and S1B Fig). Tregs were gated from the lymphocyte gate as CD4^+^CD25^+^CD127^low^ cells (S1C and S1D Fig) for analyses of Treg surface functional markers (FMO shown in S1E, S1G and S1I Fig, representative dot plots shown in F, H and J for GITR, PD-1 and CTLA-4, respectively), iTreg/nTreg ratios (unstained control shown in S1K Fig, representative dot plot of FoxP3 and Helios expression shown in L) and intracellular cytokines (FMO shown in S1M and S1O Fig, representative dot plots shown in N and P for IL-10 and TGF-β, respectively).

To confirm the validity of using Helios as a marker of nTregs by comparing its expression with another putative nTreg marker, neuropilin-1 (only several samples were stained), we stained cells with the following antibodies against surface markers: CD4 FITC (clone MEM-241, Exbio), CD25 PE (clone MEM-181, Exbio), neuropilin-1 APC/Fire750 (CD304; clone 12C2, BioLegend), followed by intracellular staining for FoxP3 APC (clone 3G3, Exbio) and Helios PE-Cy7 (clone 22F6, Exbio). TregFlowEx kit (Exbio) was used according to manufacturer’s instructions for intracellular staining and preparation of these samples.

### CFSE suppression of proliferation assay

A proliferation suppression assay was performed utilising coculture of magnetically isolated CFSE-stained target cells (non-Treg CD4^+^CD25^-^CD127^+^ cells) with magnetically isolated Treg (CD4^+^CD25^+^CD127^low^), as described previously [23,24].

EasySep™ Human CD4+CD127lowCD25+ Regulatory T Cell Isolation Kit (StemCell, Vancouver, BC, Canada) was used to magnetically isolate Tregs and target cells from cord blood mononuclear cells. The target cells were stained with 5 μM CFSE, plated into 24-well plates with or without Tregs and cultivated for 72 hours in RPMI medium (Sigma-Aldrich) supplemented with 10% FTS (Cambrex), gentamycin (Sigma-Aldrich, 40 mg/L) and L-glutamine (Sigma-Aldrich, 2mM). 20 ng of recombinant human IL-2 (PeproTech, Rocky Hill, NJ, USA), 1 μg of purified, functional grade human anti-CD3 (clone OKT3; ThermoFisherScientific) and 1 μg of purified, functional grade human anti-CD28 (clone CD28.2; ThermoFisherScientific) were added per 10^6^ target cells to stimulate proliferation. 0.5×10^6^ cells in total were seeded in each well. After 72h, cells were stained for CD4 (APC; clone MEM-241; Exbio) and analysed with flow cytometer.

### ELISA

The plasma was stored at -20°C. Levels of IL-10 and TGF-β were quantified by ELISA while specific IgE was measured immunoenzymatically by RISA (Ring-Immuno-Sorbent Assay), as described previously [25].

### Data acquisition and statistics

Flow cytometry data were acquired on a BD FACSCanto flow cytometer using BD FACS Diva version 6.1.2 software (Becton Dickinson) and analysed using FlowJo 7·2.2. (TreeStar, Ashland, OR, USA). Results of ELISA and RISA assays were obtained using Genesis software. Statistical and graphical analysis was performed using GraphPad Prism 6.0 software (GraphPad Software, La Jolla, CA, USA). Differences between groups were compared using unpaired Student’s t-test in case of data with normal distribution (total Treg, iTreg and nTreg ratios; surface markers of Treg function) and unparametric Mann-Whitney test for the rest of the data (proportions of IL-10+ Tregs and TGF-β+ Tregs, plasma levels of IL-10, TGF-β, sIgE). All results are presented as box plots with medians, 25th, and 75th percentiles as boxes and 10th and 90th percentiles as whiskers.

## Results

The immunological characteristics of cord blood of children of allergic mothers (children with high risk of allergy development, n = 108) and healthy mothers (low-risk group, n = 121) were compared. Chiefly, nTreg and iTreg populations as well as total Treg proportion were assessed. Tregs were also tested for their surface functional markers (PD-1, CTLA-4, GITR) and intracellular cytokines (IL-10, TGF-β) to reveal possible differences in functional characteristics. Such variations could contribute to the differential risk of allergy development in the two groups. Lastly, IL-10 and TGF-β levels in cord blood plasma of children as well as allergen-specific IgE levels in cord blood plasma of children and peripheral blood plasma of their mothers were tested.

### IgE

**Fig 1:**
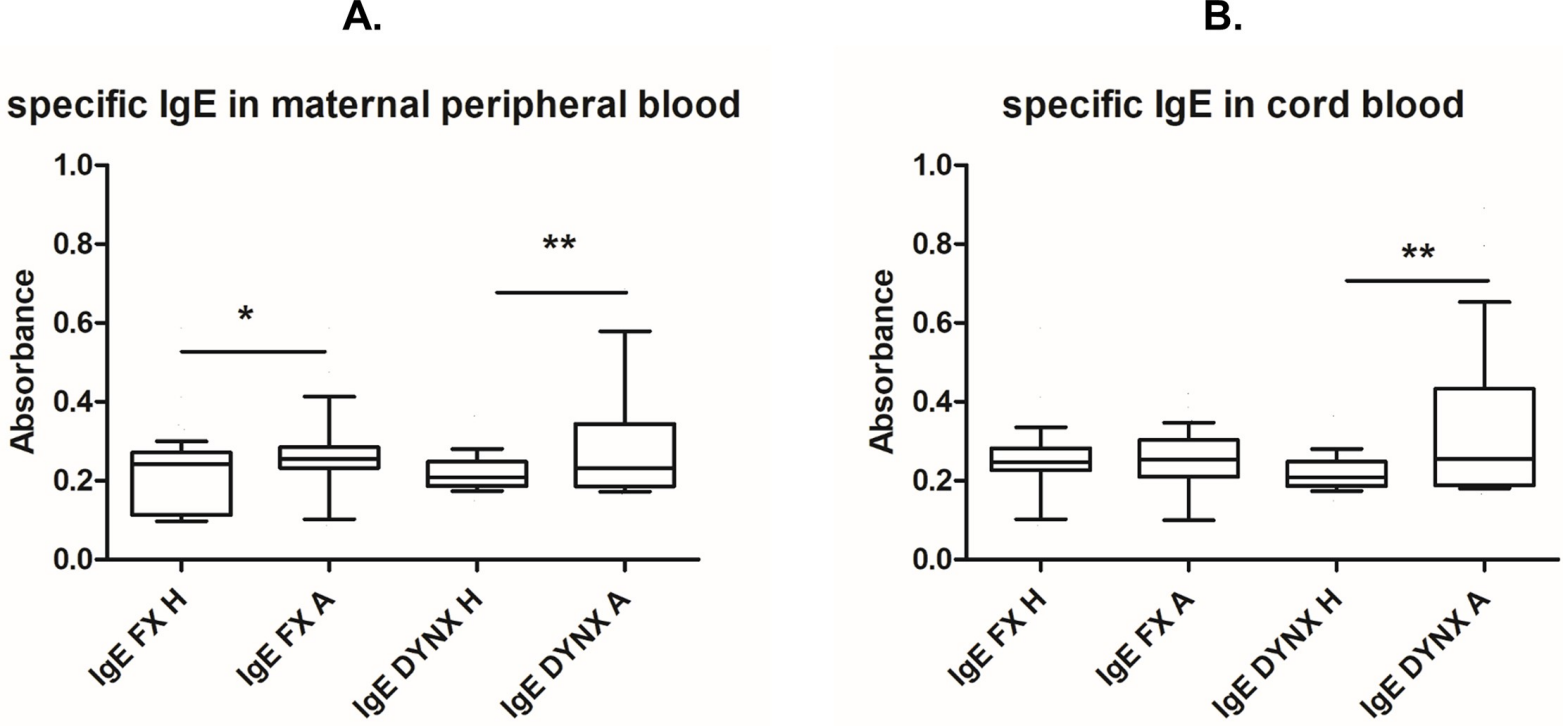
Specific IgE in maternal peripheral blood and cord blood. Samples of maternal peripheral blood and children’s cord blood of healthy (H, n = 112) and allergic (A, n = 98) mothers were collected at the time of birth. Levels of IgE specific for common food (FX) and respiratory (DYNX) allergens were determined immunoenzymatically by RISA (Ring-Immuno-Sorbent Assay). (A) Specific IgE levels in maternal peripheral blood plasma. (B) Specific IgE levels in cord blood plasma. p values were calculated using the Mann-Whitney test.

As expected, peripheral blood of allergic mothers was found to entail increased titres of IgE specific for most common food (FX, p = 0.0450) and respiratory (DYNX, p = 0.0015) allergens (Fig 1A). Increased levels of respiratory (DYNX, p = 0.0026) but not food allergen specific IgE were also detected in cord blood of children of allergic mothers (Fig 1B). This could most likely be due to transplacental transport of maternal specific IgE into fetal circulation [26].

### Treg population ratios

**Fig 2:**
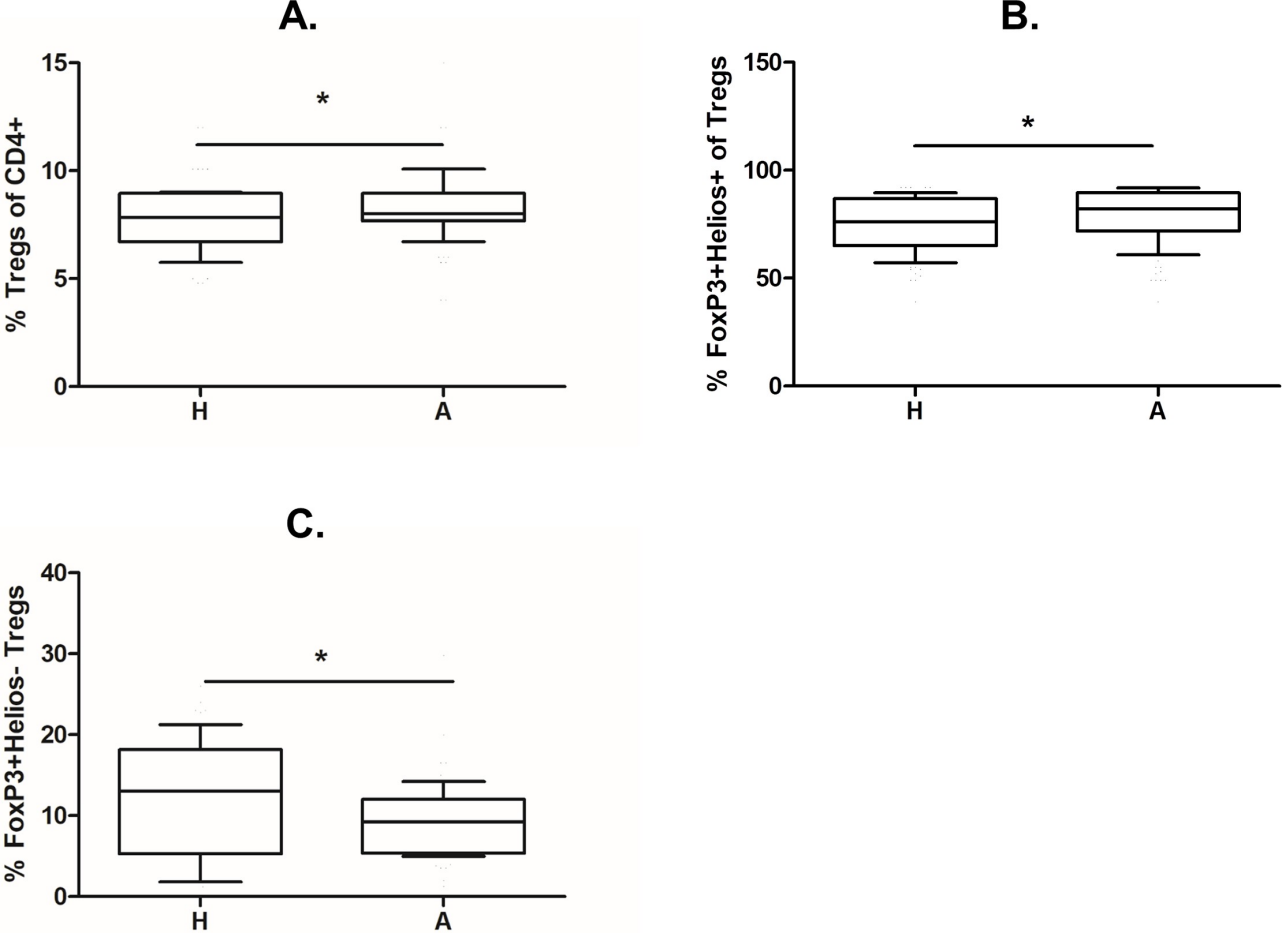
Proportions of total Treg population and nTreg and iTreg subpopulations in cord blood of children of allergic and healthy mothers. Samples of cord blood of children of healthy (H, n = 112) and allergic (A, n = 98) mothers were stained and analysed by flow cytometry. (A) Flow cytometry analysis showing the proportion of CD25^+^CD127^low^FoxP3^+^ Tregs in the cord blood CD4^+^ T cell population (p = 0.0361). (B) Flow cytometry analysis showing the proportion of FoxP3^+^Helios^+^ nTregs among CD4^+^CD25^+^CD127^low^ Tregs in cord blood (p = 0.0149). (C) Flow cytometry analysis showing the proportion of FoxP3^+^Helios^-^ iTregs among CD4^+^CD25^+^CD127^low^ Tregs in cord blood (p = 0.0175). p values were calculated using unpaired Student’s t-test.

Using our gating strategy and a combination of surface and intracellular markers, we observed a significantly increased proportion of CD25^+^CD127^low^FoxP3^+^ Tregs in CD4^+^ T cells from the cord blood of children of allergic mothers (Fig 2A; p = 0.0361). After adding antibodies against Helios, a transcription factor characteristic for natural but not induced Tregs, we revealed a higher proportion of FoxP3^+^Helios^+^ nTregs (Fig 2B; p = 0.0149) and a lower proportion of FoxP3^+^Helios^-^ iTregs (Fig 2C; p = 0.0175) in CD4^+^CD25^+^CD127^low^ Tregs of children of allergic mothers. We have tried to stain several samples for neuropilin-1, another putative marker of nTregs, in addition to Helios. Helios positive fraction of CD25^high^FoxP3^+^ Tregs expressed neuropilin at higher MFI compared with Helios negative cells (S2 Fig). Nevertheless, we also observed numerous Helios^+^neuropilin^-^ Treg, suggesting different dynamics and/or roles of these markers in the context of human regulatory T cells.

### Treg functional markers

**Fig 3:**
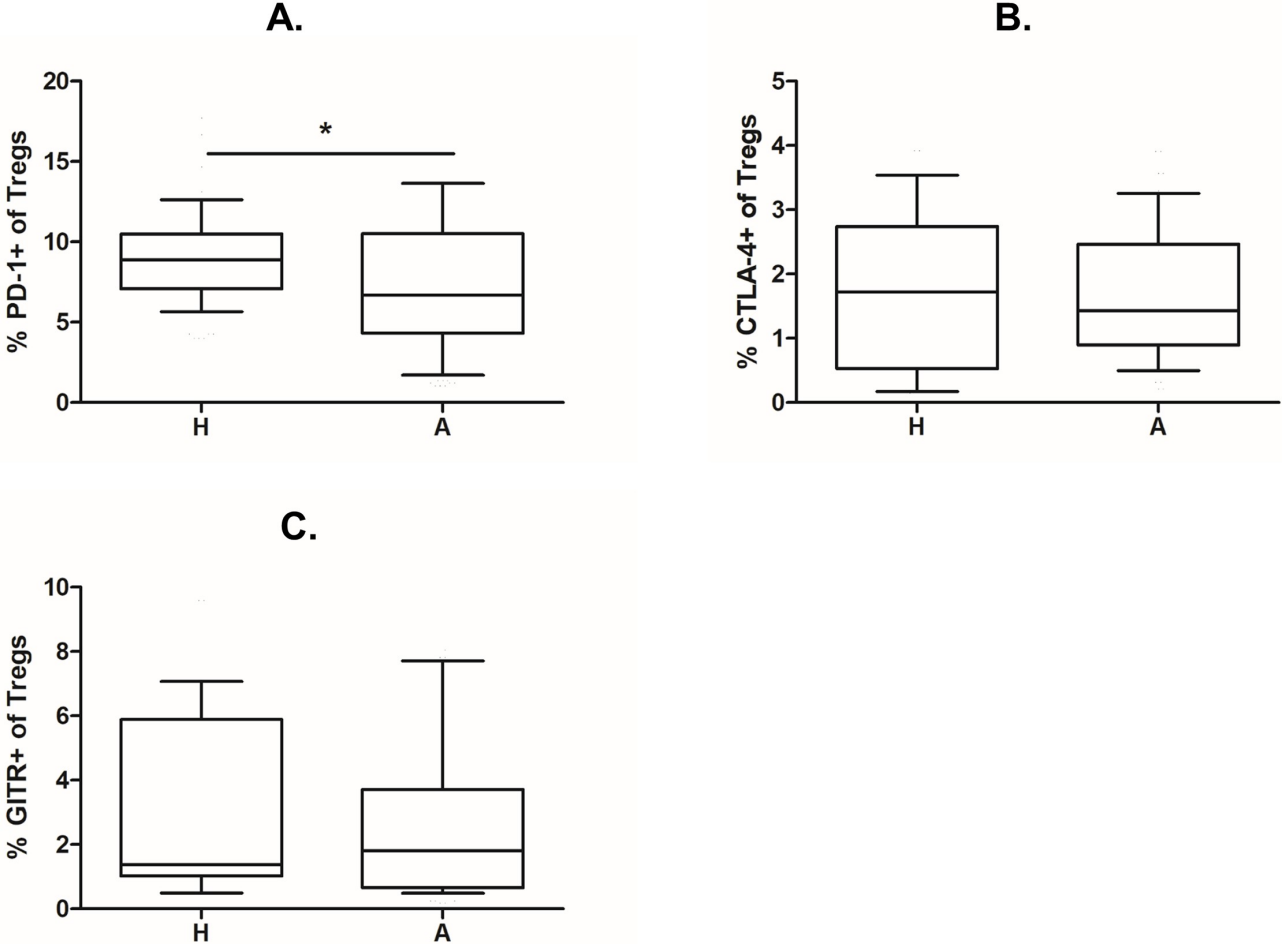
Expression of surface markers of Treg function in Tregs from cord blood of children of allergic and healthy mothers. Samples of cord blood of children of healthy (H, n = 112) and allergic (A, n = 98) mothers were stained and analysed by flow cytometry. (A) Flow cytometry analysis showing the proportion of PD-1^+^ cells among CD4^+^CD25^+^CD127^low^ Tregs in cord blood (p = 0.0382). (B) Flow cytometry analysis showing the proportion of CTLA-4^+^ cells among CD4^+^CD25^+^CD127^low^ Tregs in cord blood. (C) Flow cytometry analysis showing the proportion of GITR^+^ cells among CD4^+^CD25^+^CD127^low^ Tregs in cord blood. p values were calculated using unpaired Student’s t-test.

To compare the expression of functional parameters, Treg surface markers of function CTLA-4, PD-1 and GITR were stained and evaluated as a percentage of CD4^+^CD25^+^CD127^low^ Treg cells. A significantly higher proportion of PD-1^+^ Tregs was found in cord blood of children of healthy mothers (Fig 3A; p = 0.0382). A similar trend, albeit not significant, can be seen for CTLA^+^ Tregs (Fig 3B) and to a smaller extent also GITR^+^ Tregs (Fig 3C).

**Fig 4:**
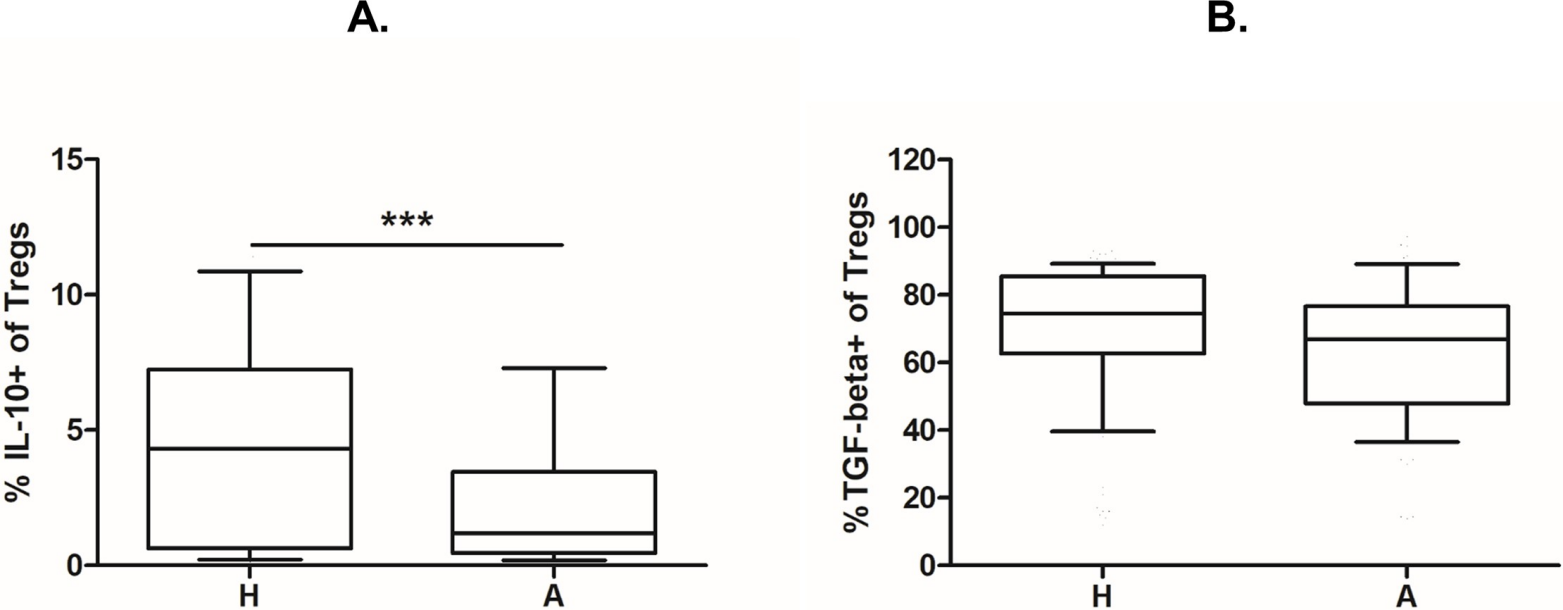
Intracellular expression of regulatory cytokines in Tregs from cord blood of children of allergic and healthy mothers. Samples of cord blood of children of healthy (H, n = 112) and allergic (A, n = 98) mothers were stained and analysed by flow cytometry for intracellular presence of cytokines IL-10 and TGF-β. (A) Flow cytometry analysis showing the proportion of IL-10^+^ cells among CD4^+^CD25^+^CD127^low^ Tregs in cord blood (p = 0. 0006). (B) Flow cytometry analysis showing the proportion of TGF-β^+^ cells among CD4^+^CD25^+^CD127^low^ Tregs in cord blood. p values were calculated using the Mann-Whitney test.

Furthermore, intracellular presence of regulatory cytokines IL-10 and TGF-β was assessed using intracellular staining. The proportion of IL-10 and TGF-beta positive Tregs was analysed as described previously [22]. A significantly higher percentage of IL-10^+^ Tregs was found in cord blood of children of healthy mothers compared with children of allergic mothers (Fig 4A; p = 0.0006). The same trend is discernible for TGF-β, although it is not significant (Fig 4B).

### Treg functional assay

To further assess functional ability of Tregs from cord blood of newborns of allergic and healthy mothers, proliferation suppression assay based on coculture of CFSE-stained target cells (non-Treg CD4^+^CD25^-^CD127^+^ cells) with Tregs was performed on several samples (n = 19). Although the number of samples we were able to include was insufficient for proper statistical analysis, we observed a trend of impaired ability of Treg isolated from cord blood to suppress proliferation of target cells in comparison to Treg from peripheral blood of adults (S3C Fig). The suppressive function was notably less effective in Tregs isolated from cord blood of children of allergic mothers (S3B Fig) compared to newborns of healthy mothers (S3A Fig). A summary table with the percentage of proliferating cells and the number of cell divisions (S1 Table) shows extreme individual variability, which may be related to the inherent variability of cord blood in different clinical context of an individual pregnancy. Another factor may be the limited number of samples analysed. Additional studies would be warranted to confirm if distinct patterns of cord blood cells in the data could be traced by correlation with subsequent allergy development.

### Regulatory cytokine levels in cord blood

**Fig 5:**
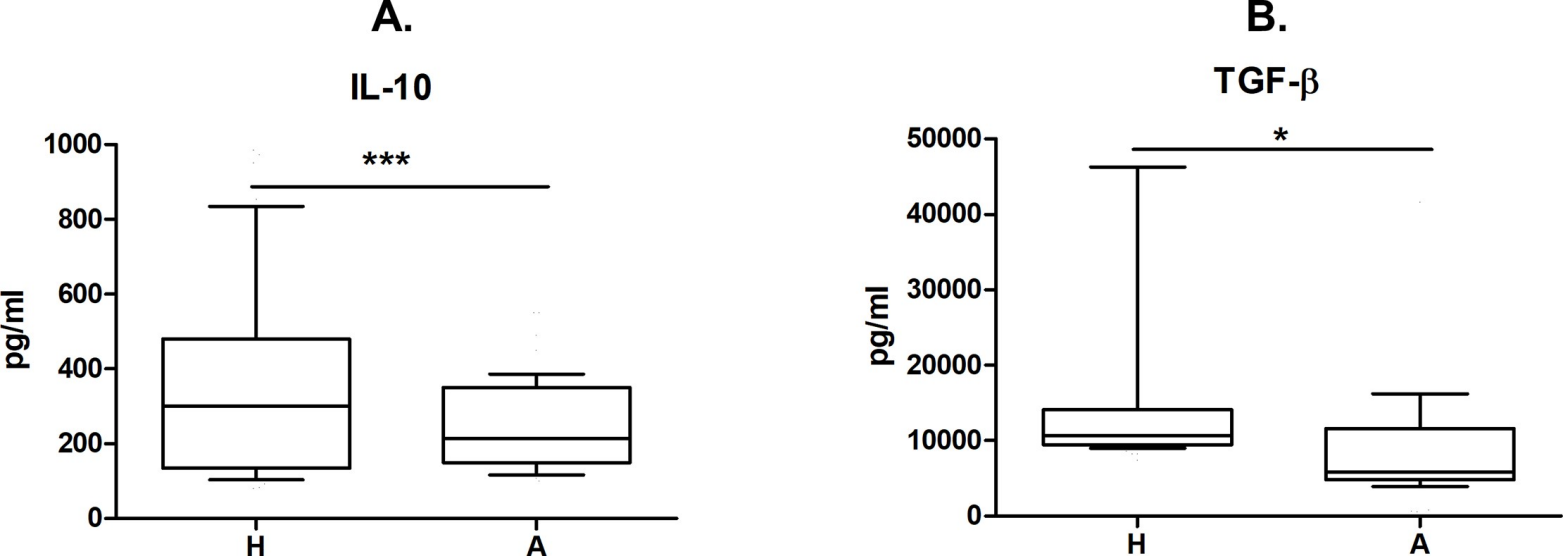
Regulatory cytokine levels in cord blood of children of allergic and healthy mothers. Samples of cord blood plasma of children of healthy (H, n = 112) and allergic (A, n = 98) mothers were collected at the time of birth. Levels of IL-10 (A, p = 0.0009) and TGF-β (B, p = 0. 0492) in plasma were analysed by ELISA. p values were calculated using the Mann-Whitney test.

In order to verify if the differences of immune regulation extend to the systemic level, IL-10 and TGF-β concentration in cord blood plasma was determined by ELISA. Significantly higher levels of both IL-10 and TGF-β were found in cord blood of children of healthy mothers than in the high-risk group of children of allergic mothers (Fig 5; p = 0.0009 and 0.0492, respectively).

## Discussion

Regulatory T cells play a key role in establishment and maintenance of balance among different branches of the immune system both prenatally and postnatally [19,27]. Nevertheless, the exact mechanisms of Treg involvement in early stages of sensitization and allergy development have not been conclusively elucidated.

We found out differences between function-associated parameters and subpopulation characteristics of Tregs from cord blood of children of allergic mothers compared with children of healthy mothers. The decrease in some of the functional markers (PD-1, IL-10) points to a lower functional efficacy of Tregs from new-borns of allergic mothers [6]. This could be partially compensated by the increased number of Tregs in comparison with children of healthy mothers. Lower numbers of Helios-FoxP3+ Tregs, putative iTregs, in cord blood from children of allergic mothers may be another sign implying abnormal or delayed Treg development contributing to the increased risk of allergy development in these children. This seems to be in accordance with our current findings of decreased cord blood levels of regulatory cytokines IL-10 and TGF-β as well as higher cord blood levels of specific IgE in children of allergic mothers compared with children of healthy mothers.

Children of allergic mothers exhibited an increased proportion of CD4^+^CD25^+^CD127^low^FoxP3^+^ Tregs. This finding is consistent with our work published earlier [7] as well as with the data from some other groups [28,29], while some studies described no significant difference [30] or even observed an opposite trend [31]. Several factors may account for these discrepancies. Although identification of human Tregs as CD25^+^CD127^low^FoxP3^+^ population within CD4^+^ cells is now more or less commonly accepted, different gating strategies have previously been used for Treg identification, and slightly different population proportions were thus obtained based on gating hierarchy and markers used [7,28]. This can be further pronounced when different monoclonal antibody clones are used in flow cytometry [32]. Another important point is that beside bona fide Tregs (CD4^+^CD25^+^CD127^low^FoxP3^+^), activated effector T cells also upregulate CD25 expression [33]. CD25^high^ population (approx. top 2% of expression) has been shown to correlate better with FoxP3 expression and regulatory phenotype [34], probably limiting the contamination of studied Treg sample with activated effector cells. FoxP3 transcription factor, previously thought exclusive for committed Tregs, has been described to be transiently upregulated upon activation of effector T cells [35]. Moreover, considering Tregs as only CD4+CD25+ [36] could lead to overestimation of Tregs especially in the group of newborns of allergic mothers. As we have shown, increased proliferation of cord blood cells [37] and CD25 is commonly accepted as a marker associated with activation. Epigenetic markers such as Treg-specific demethylated region (TSDR) are currently being proposed as better correlating with Treg commitment and functional activity [38].

Tregs are quite a diverse cell population with some degree of plasticity and numerous described subpopulations with varying functional roles in the context of different clinical settings and tissues [39,40]. The development of these subpopulations as well as regulatory capabilities is influenced by factors both intrinsic and external [19,20], including maternal health status, lifestyle and in utero exposure to microbial antigens, allergens or other environmental factors [19]. This could further compromise the comparability of data from different studies, specifically those carried out in rural [19,27,28] vs. urban environment [29].

Of possible importance in the context of allergy development is the proposed difference between thymus-derived natural Tregs (nTregs) and a peripheral population of induced Tregs (iTregs) [8]. There is some controversy regarding the identification of these populations, and no reliable, indisputable distinguishing markers have been established so far. Helios, an Ikaros-family transcription factor, has been proposed as a marker specific for thymic-derived Tregs, i. e. nTregs, in humans [9] in conjunction with FoxP3 expression. We observed significantly higher proportion of FoxP3^+^ Helios^-^ iTregs in Tregs from cord blood of children of healthy mothers compared with the allergic group. Children of allergic mothers, in comparison, had higher proportion of FoxP3^+^ Helios^+^ nTregs in Treg population. Since iTregs are induced in periphery upon recognition of harmless environmental antigens under tolerogenic conditions, we assume they may play a vital role in downregulating inappropriate immune responses towards allergens. The lower percentage of iTregs among Tregs of children of allergic mothers may be a factor compromising early postnatal capacity of the immune system to establish tolerance to harmless environmental antigens. As these cells mainly arise upon exposure to environmental influences [12], i.e. later in the process of perinatal immune system maturation, we hypothesise that their relatively lower levels might imply a less mature immune system at birth in the group with higher relative risk of allergy development. To the best of our knowledge, these findings present the first published data regarding Helios expression in cord blood Tregs in the context of allergy.

The suitability of Helios as a marker specific for nTregs has been questioned by a number of studies performed in mice [41,42] as well as several studies involving human subjects [43,44]. Helios^-^ cells have been found within the population of CD45RA+FoxP3+ Tregs, suggesting that antigen-naïve presumed nTregs may not universally exhibit the Foxp3+ Helios+ expression pattern [44]. Likewise, Helios expression has been described in induced Tregs and even FoxP3- CD4+ and CD8+ cells [43]. Different explanations of the role of Helios have consequently been proposed.

It may be possible that Helios expression rises after exposure of the cell to cognate antigen and Helios may thus serve as an activation and proliferation marker [43], similar to FoxP3 itself [33]. The higher proportion of Helios+ FoxP3+ cells in unstimulated cord blood of the children of allergic mothers could then be viewed as a sign of generally higher reactivity of cells derived from cord blood of this group; these results are consistent with studies previously published by our group in this context [17,45–47]. This higher reactivity of cells in cord blood of the high-risk group may signify an immune milieu more prone to inflammation, including allergy.

Another hypothesis put forward is that Helios may play a role in Treg development and the establishment of a stable regulatory phenotype [48]. In such case, the higher expression of Helios we observed may in fact reflect the compensatory expansion of the Treg population brought about by their compromised function. Alternatively, we hypothesise that the lower maturity of the immune system of high-risk children at birth might conceivably mean that fewer stable, lineage-committed Tregs (with no further need of Helios expression) are present at birth. Again, this could also contribute to the higher risk of allergy development in these children.

To help contend with the challenge inherent in distinguishing iTregs from nTregs, we introduced staining for neuropilin-1, another proposed marker of nTregs [11]. Although the number of samples analysed was not sufficient for statistical analysis, we observed a slightly higher expression of neuropilin-1 expression on Helios+ Tregs, a trend consistent with both being considered putative nTreg markers [10]. As neuropilin-1 has been described to play a role in Treg stability maintenance [49], the mechanisms involved in its regulation may also be similar to those discussed earlier in the case of Helios. The full significance of neuropilin-1 expression on circulating human regulatory T cells is not yet clear, however, since most convincing studies were either performed in mice [11] or concerned with the context of lymph node resident Tregs [50].

In performing their regulatory functions, Tregs employ both secreted molecules (chiefly regulatory cytokines IL-10 and TGF-β) and surface proteins. In our study, we observed a significantly lower proportion of PD-1^+^ Tregs in children of allergic mothers. The same trend is also hinted at for CTLA-4 and GITR, though the statistical power was insufficient for these smaller differences to be significant. PD-1 and CTLA-4 are two of the most ubiquitous Treg functional markers, serving as important coinhibitory molecules involved in cell to cell communication between Tregs and target cells [51], the so called immune checkpoints. They play a crucial role in regulation of autoimmune diseases [52,53] and allergy [54,55]. CTLA-4 involvement has been reported in food allergy [55] as well as the establishment of Th1 and Th2 balance [56], while PD-1 expression by Tregs was shown to play a major role in an animal model of antigen-induced asthma [57].

Targeting these molecules for inhibition also forms the basis of highly effective novel antitumor therapy–the “checkpoint blockade” [58]; the high incidence of immune-related adverse effects accompanying this treatment modality attests to their pronounced functional relevance [59]. GITR is likewise generally considered a marker of Treg function [60], albeit less well-defined functionally and likely serving a negative feedback role in the regulation of Treg homeostasis [61]. The lower expression of PD-1 and other surface markers in Tregs from cord blood of children of allergic mothers marks decreased functional ability of these cells, which in turn could contribute to increased risk of allergy development.

Children of allergic mothers also showed a significantly lower number of IL-10^+^ Tregs, with the same trend visible for TGF-β. IL-10 and TGF-β are the two chief regulatory cytokines and have been proven to play crucial roles in controlling allergy and establishing tolerance to environmental antigens [62–64]. IL-10 production critically contributes to in vivo suppressive efficacy of Tregs [6], and IL-10 producing CD4 cells, termed Tr1 cells, are implicated in the success of allergen-specific immune therapy [5]. TGF-β is essential for establishing and maintaining tolerance to harmless environmental (e.g. microbial) antigens in the mucosal tissues [65], iTreg induction in the periphery [12,66] and the fine tuning of the mucosal immune system [67,68]. The decrease in intracellular IL-10 as well as lower plasma levels observed in allergic children are in keeping with our previously published observations–higher proliferation activity of CD4+ T cells [69] and increased activation of dendritic cells [47] isolated from cord blood of children of allergic mothers.

To assess the suppressive capacity of cord blood Tregs directly, we also performed a proliferation inhibition assay. Although the data is only preliminary so far, we could demonstrate a notable deficiency of suppressive function of Tregs isolated from cord blood compared with Tregs isolated from adult peripheral blood. This deficiency was more pronounced in Tregs from cord blood of a newborn of allergic mother compared with Tregs from cord blood of a child of a healthy mother. Although these findings need confirmation with further experiments, they could support our proposed hypothesis regarding functional maturity of Tregs of children of allergy mothers.

Impaired Treg function, evidenced by lower functional marker expression, as well as smaller number of induced Tregs in these children could compromise the capacity of their immune systems to correctly set up the immune system balance in early postnatal period. Perinatal period is critical for this fine tuning as the organism first encounters numerous environmental factors, including microbial antigens, as well as potential food and respiratory antigens. Taken together, our results imply that the immune system of children of allergic mothers tends to be more immature at the time of birth, and therefore more prone to developing dysbalance such as allergy. The increased proportion of total Tregs seen in these children is then likely an attempt of the system to compensate for the lower functional capabilities by upregulating the population size. Nevertheless, several points still need to be addressed. Since the flow cytometric analysis of intracellular cytokines and surface markers presents an indirect view of Treg suppressive efficiency, these observations need to be confirmed by functional assays. Preliminary data shown in the article support positive correlation between impaired production of immunoregulatory cytokines and lower capacity to limit proliferation of effector CD4+ T cells. Nevertheless, this needs to be confirmed on a larger scale to bring definitive insight into the suggested difference of Treg functional capacity between high-risk and low-risk groups. In addition to that, our data suggest that neonatal Tregs have a lower immunosuppressive capacity in comparison to adult ones. There is also a pressing need of further research into markers characteristic for natural and induced Tregs. Presently, there are no universally accepted markers capable of reliably distinguishing the two populations, greatly complicating any analysis of their respective importance in different contexts. The conundrum regarding the role and function of Helios within the context of nTreg and iTreg subpopulations needs to be resolved before a conclusive picture of its possible significance for allergy prediction can be drawn. Lastly, long-term prospective monitoring of children will be necessary for assessment of the predictive value of individual findings and perinatal Treg insufficiency as a whole for actual future development of allergy.

## Supporting information

S1 Fig Gating strategy, FMO controls and representative dot plots of surface and intracellular markers. Cord blood samples were stained and analysed by flow cytometry. A-D: Gating strategy of CD4^+^CD25^+^CD127^low^ cells.E-P: FMO controls and representative dot plots of CD4^+^CD25^+^CD127^low^ cells stained for surface (E-J) and intracellular (K-P) markers. E, G, I: FMO controls for GITR, PD-1 and CTLA-4 staining. F, H, J: Representative dot plots of GITR, PD-1, CTLA-4. K: Control sample unstained for FoxP3 and Helios. L: Representative dot plot of intracellular staining for FoxP3 and Helios. M, O: FMO control for intracellular staining of IL-10 and TGF-β. N, P: Representative dot plot of intracellular staining for IL-10 and TGF-β.
(TIF)

S2 Fig Representative histograms of staining for neuropilin-1. Several whole blood samples were stained for CD4, CD25, neuropilin-1, FoxP3 and Helios and analysed by flow cytometry. A: CD25^high^FoxP3^+^ cells were gated into Helios^+^ and Helios^-^ populations. B: Expression of neuropilin-1 on Helios^+^ (blue) and Helios^-^ (red) cells. FMO control for neuropilin-1 shown in black.
(TIF)

S3 Fig Suppression of proliferation of CFSE-stained non-Treg cells. CD4^+^CD25^-^CD127^+^ target cells were magnetically isolated from cord blood mononuclear cells (n = 19), stained with 5 μM CFSE and cocultured with CD4^+^CD25^+^CD127^low^ Treg cells at 1:5 Treg:target cell ratio. After 72 hours, cells were harvested, stained for CD4 and analysed by flow cytometry. Representative histograms show unstimulated control cells (blue), anti-CD3/CD28 stimulated control cells (red) and stimulated cells cocultured with Tregs at 1:5 Treg:target ratio (orange). A: Cells isolated from cord blood of a newborn of a healthy mother. B: Cells isolated from cord blood of a newborn of an allergic mother. C: Cells isolated from adult peripheral blood.
(TIF)

S1 Table Summary table of data from CFSE-based suppression assays. CD4^+^CD25^-^CD127^+^ target cells were magnetically isolated from cord blood mononuclear cells (n = 19), stained with 5 μM CFSE and cocultured with CD4^+^CD25^+^CD127^low^ Treg cells at 1:5 Treg:target cell ratio. After 72 hours, cells were harvested, stained for CD4 and analysed by flow cytometry. Table shows percentage of cells which went through at least one round of cell division (Divided cells), percentage of cells which did not proliferate (Undivided cells) and the number of peaks representing cell divisions in each sample (Number of generations). For each sample, allergy status is shown (A–children of allergic mothers, H–children of healthy mothers) and three conditions are included: Tregs cocultured with target cells at 1:5 Treg:target ratio; target cells stimulated with CD3 and CD28 monoclonal antibodies and IL-2; and unstimulated target cells, with only IL-2 added.
(PDF)

## References

[1] Sykes L, MacIntyre DA, Yap XJ, Teoh TG, Bennett PR. The Th1:th2 dichotomy of pregnancy and preterm labour. Mediators Inflamm. 2012;2012:967629 10.1155/2012/967629 22719180 10.1155/2012/967629 PMC3376783

[2] Jutel M, Akdis CA. T-cell Subset Regulation in Atopy. Curr Allergy Asthma Rep. 2011 4;11(2):139–45. 10.1007/s11882-011-0178-7 21271314 10.1007/s11882-011-0178-7 PMC3047206

[3] Steinborn A, Engst M, Haensch GM, Mahnke K, Schmitt E, Meuer S, et al Small for gestational age (SGA) neonates show reduced suppressive activity of their regulatory T cells. Clin Immunol Orlando Fla. 2010 2;134(2):188–97. 10.1016/j.clim.2009.09.003 19837002

[4] Bacchetta R, Passerini L, Gambineri E, Dai M, Allan SE, Perroni L, et al Defective regulatory and effector T cell functions in patients with FOXP3 mutations. J Clin Invest. 2006 6;116(6):1713–22. 10.1172/JCI25112 16741580 10.1172/JCI25112 PMC1472239

[5] Akdis CA, Akdis M. Mechanisms of allergen-specific immunotherapy. J Allergy Clin Immunol. 2011 1;127(1):18–27; quiz 28–9. 10.1016/j.jaci.2010.11.030 21211639 10.1016/j.jaci.2010.11.030

[6] Shevach EM. Mechanisms of Foxp3+ T Regulatory Cell-Mediated Suppression. Immunity. 2009 5;30(5):636–45. 10.1016/j.immuni.2009.04.010 19464986 10.1016/j.immuni.2009.04.010

[7] Hrdý J, Kocourková I, Prokešová L. Impaired function of regulatory T cells in cord blood of children of allergic mothers: Tregs in cord blood and allergy risk. Clin Exp Immunol. 2012 10;170(1):10–7. 10.1111/j.1365-2249.2012.04630.x 22943196 10.1111/j.1365-2249.2012.04630.x PMC3444712

[8] Shevach EM, Thornton AM. tTregs, pTregs, and iTregs: similarities and differences. Immunol Rev. 2014 5;259(1):88–102. 10.1111/imr.12160 24712461 10.1111/imr.12160 PMC3982187

[9] Thornton AM, Korty PE, Tran DQ, Wohlfert EA, Murray PE, Belkaid Y, et al Expression of Helios, an Ikaros transcription factor family member, differentiates thymic-derived from peripherally induced Foxp3+ T regulatory cells. J Immunol Baltim Md 1950. 2010 4 1;184(7):3433–41. 10.4049/jimmunol.0904028 PMC3725574 20181882

[10] Singh K, Hjort M, Thorvaldson L, Sandler S. Concomitant analysis of Helios and Neuropilin-1 as a marker to detect thymic derived regulatory T cells in naïve mice. Sci Rep. 2015 1 14;5:7767 10.1038/srep07767 25586548 10.1038/srep07767 PMC4293597

[11] Weiss JM, Bilate AM, Gobert M, Ding Y, Curotto de Lafaille MA, Parkhurst CN, et al Neuropilin 1 is expressed on thymus-derived natural regulatory T cells, but not mucosa-generated induced Foxp3+ T reg cells. J Exp Med. 2012 9 24;209(10):1723–42, S1. 10.1084/jem.20120914 22966001 10.1084/jem.20120914 PMC3457733

[12] Chen W, Jin W, Hardegen N, Lei K-J, Li L, Marinos N, et al Conversion of peripheral CD4+CD25- naive T cells to CD4+CD25+ regulatory T cells by TGF-beta induction of transcription factor Foxp3. J Exp Med. 2003 12 15;198(12):1875–86. 10.1084/jem.20030152 14676299 10.1084/jem.20030152 PMC2194145

[13] Prescott SL, King B, Strong TL, Holt PG. The value of perinatal immune responses in predicting allergic disease at 6 years of age. Allergy. 2003 11;58(11):1187–94. 14616132 10.1034/j.1398-9995.2003.00263.x

[14] Peters JL, Cohen S, Staudenmayer J, Hosen J, Platts-Mills TAE, Wright RJ. Prenatal negative life events increases cord blood IgE: interactions with dust mite allergen and maternal atopy. Allergy. 2012 4;67(4):545–51. 10.1111/j.1398-9995.2012.02791.x 22309645 10.1111/j.1398-9995.2012.02791.x PMC3303977

[15] Prokesová L, Novotná O, Janatková I, Zanvit P, Zizka J, Lodinová-Zádníková R, et al IgE against food and respiratory allergens in healthy and allergic mothers and their children. Folia Microbiol (Praha). 2008;53(1):67–72. 18481221 10.1007/s12223-008-0010-5

[16] Chung EK, Miller RL, Wilson MT, McGeady SJ, Culhane JF. Antenatal risk factors, cytokines and the development of atopic disease in early childhood. Arch Dis Child—Fetal Neonatal Ed. 2007 1 1;92(1):F68–73. 10.1136/adc.2006.106492 17185433 10.1136/adc.2006.106492 PMC2675311

[17] Hrdý J, Zanvit P, Novotná O, Kocourková I, Žižka J, Prokešová L. Cytokine expression in cord blood cells of children of healthy and allergic mothers. Folia Microbiol (Praha). 2010 9;55(5):515–9. 20941589 10.1007/s12223-010-0085-7

[18] Rindsjö E, Joerink M, Johansson C, Bremme K, Malmström V, Scheynius A. Maternal allergic disease does not affect the phenotype of T and B cells or the immune response to allergens in neonates: No effect of maternal allergy on neonatal lymphocytes. Allergy. 2009 11 23;65(7):822–30. 10.1111/j.1398-9995.2009.02266.x 19930231 10.1111/j.1398-9995.2009.02266.x

[19] Schaub B, Liu J, Höppler S, Schleich I, Huehn J, Olek S, et al Maternal farm exposure modulates neonatal immune mechanisms through regulatory T cells. J Allergy Clin Immunol. 2009 4;123(4):774–782.e5. 10.1016/j.jaci.2009.01.056 19348917 10.1016/j.jaci.2009.01.056

[20] Schröder PC, Casaca VI, Illi S, Schieck M, Michel S, Böck A, et al IL-33 polymorphisms are associated with increased risk of hay fever and reduced regulatory T cells in a birth cohort. Pediatr Allergy Immunol Off Publ Eur Soc Pediatr Allergy Immunol. 2016 11;27(7):687–95. 10.1111/pai.12597 27171815

[21] Meng S, Gao R, Yan B, Ren J, Wu F, Chen P, et al Maternal allergic disease history affects childhood allergy development through impairment of neonatal regulatory T-cells. Respir Res [Internet]. 2016 12 [cited 2017 Jan 31];17(1). Available from: http://respiratory-research.biomedcentral.com/articles/10.1186/s12931-016-0430-8 10.1186/s12931-016-0430-8 PMC5028930 27646403

[22] Hrdý J, Kocourková I, Lodinová-Žádníková R, Kolářová L, Prokešová L. The effect of a probiotic Escherichia coli strain on regulatory T-cells in six year-old children. Benef Microbes. 2016 11 30;7(5):639–48. 10.3920/BM2016.0030 27633175 10.3920/BM2016.0030

[23] Strauss L, Whiteside TL, Knights A, Bergmann C, Knuth A, Zippelius A. Selective survival of naturally occurring human CD4+CD25+Foxp3+ regulatory T cells cultured with rapamycin. J Immunol Baltim Md 1950. 2007 1 1;178(1):320–9. 10.4049/jimmunol.178.1.320 17182569

[24] Strauss L, Czystowska M, Szajnik M, Mandapathil M, Whiteside TL. Differential Responses of Human Regulatory T Cells (Treg) and Effector T Cells to Rapamycin. Unutmaz D, editor. PLoS ONE. 2009 6 22;4(6):e5994 10.1371/journal.pone.0005994 19543393 10.1371/journal.pone.0005994 PMC2694984

[25] Lodinová-Žádníková R, Prokešová L, Kocourková I, Hrdý J, Žižka J. Prevention of Allergy in Infants of Allergic Mothers by Probiotic Escherichia coli. Int Arch Allergy Immunol. 2010;153(2):201–6. 10.1159/000312638 20413988 10.1159/000312638

[26] Bundhoo A, Paveglio S, Rafti E, Dhongade A, Blumberg RS, Matson AP. Evidence that FcRn mediates the transplacental passage of maternal IgE in the form of IgG anti-IgE/IgE immune complexes. Clin Exp Allergy J Br Soc Allergy Clin Immunol. 2015 6;45(6):1085–98. 10.1111/cea.12508 PMC4437844 25652137

[27] Schaub B, Liu J, Schleich I, Höppler S, Sattler C, von Mutius E. Impairment of T helper and T regulatory cell responses at birth. Allergy. 2008 11;63(11):1438–47. 10.1111/j.1398-9995.2008.01685.x 18925880 10.1111/j.1398-9995.2008.01685.x

[28] Strömbeck A, Rabe H, Lundell A-C, Andersson K, Johansen S, Adlerberth I, et al High proportions of FOXP3 ^+^ CD25 ^high^ T cells in neonates are positively associated with allergic sensitization later in childhood. Clin Exp Allergy. 2014 7;44(7):940–52. 10.1111/cea.12290 24528482 10.1111/cea.12290 PMC4215110

[29] McLoughlin RM, Calatroni A, Visness CM, Wallace PK, Cruikshank WW, Tuzova M, et al Longitudinal relationship of early life immunomodulatory T cell phenotype and function to development of allergic sensitization in an urban cohort. Clin Exp Allergy J Br Soc Allergy Clin Immunol. 2012 3;42(3):392–404. 10.1111/j.1365-2222.2011.03882.x PMC4162345 22092655

[30] Fu Y, Lou H, Wang C, Lou W, Wang Y, Zheng T, et al T cell subsets in cord blood are influenced by maternal allergy and associated with atopic dermatitis. Pediatr Allergy Immunol. 2013 3;24(2):178–86. 10.1111/pai.12050 23506292 10.1111/pai.12050

[31] Hinz D, Bauer M, Röder S, Olek S, Huehn J, Sack U, et al Cord blood Tregs with stable FOXP3 expression are influenced by prenatal environment and associated with atopic dermatitis at the age of one year. Allergy. 2012 3;67(3):380–9. 10.1111/j.1398-9995.2011.02767.x 22187950 10.1111/j.1398-9995.2011.02767.x

[32] Law JP, Hirschkorn DF, Owen RE, Biswas HH, Norris PJ, Lanteri MC. The importance of Foxp3 antibody and fixation/permeabilization buffer combinations in identifying CD4+CD25+Foxp3+ regulatory T cells. Cytom Part J Int Soc Anal Cytol. 2009 12;75(12):1040–50. 10.1002/cyto.a.20815 PMC2862733 19845018

[33] Kmieciak M, Gowda M, Graham L, Godder K, Bear HD, Marincola FM, et al Human T cells express CD25 and Foxp3 upon activation and exhibit effector/memory phenotypes without any regulatory/suppressor function. J Transl Med. 2009;7(1):89. 19849846 10.1186/1479-5876-7-89 PMC2770477

[34] Roncador G, Brown PJ, Maestre L, Hue S, Martínez-Torrecuadrada JL, Ling K-L, et al Analysis of FOXP3 protein expression in human CD4+CD25+ regulatory T cells at the single-cell level. Eur J Immunol. 2005 6;35(6):1681–91. 10.1002/eji.200526189 15902688 10.1002/eji.200526189

[35] Allan SE, Crome SQ, Crellin NK, Passerini L, Steiner TS, Bacchetta R, et al Activation-induced FOXP3 in human T effector cells does not suppress proliferation or cytokine production. Int Immunol. 2007 2 20;19(4):345–54. 10.1093/intimm/dxm014 17329235 10.1093/intimm/dxm014

[36] Akdis CA, Akdis M. Mechanisms of allergen-specific immunotherapy and immune tolerance to allergens. World Allergy Organ J. 2015 12;8(1):1–12. 10.1186/1939-4551-8-1 26023323 10.1186/s40413-015-0063-2 PMC4430874

[37] Zizka J, Hrdý J, Lodinová-Zádníková R, Kocourková I, Novotná O, Sterzl I, et al Effect of breast milk of healthy and allergic mothers on in vitro stimulation of cord blood lymphocytes. Pediatr Allergy Immunol Off Publ Eur Soc Pediatr Allergy Immunol. 2007 9;18(6):486–494. 10.1111/j.1399-3038.2007.00563.x 17651385

[38] Kitagawa Y, Ohkura N, Sakaguchi S. Epigenetic control of thymic Treg-cell development. Eur J Immunol. 2015 1;45(1):11–6. 10.1002/eji.201444577 25348287 10.1002/eji.201444577

[39] Campbell DJ, Koch MA. Phenotypical and functional specialization of FOXP3+ regulatory T cells. Nat Rev Immunol. 2011 2;11(2):119–30. 10.1038/nri2916 21267013 10.1038/nri2916 PMC3289970

[40] Wing JB, Sakaguchi S. Multiple treg suppressive modules and their adaptability. Front Immunol. 2012;3:178 10.3389/fimmu.2012.00178 22754556 10.3389/fimmu.2012.00178 PMC3386489

[41] Gottschalk RA, Corse E, Allison JP. Expression of Helios in peripherally induced Foxp3+ regulatory T cells. J Immunol Baltim Md 1950. 2012 2 1;188(3):976–80. 10.4049/jimmunol.1102964 22198953

[42] Szurek E, Cebula A, Wojciech L, Pietrzak M, Rempala G, Kisielow P, et al Differences in Expression Level of Helios and Neuropilin-1 Do Not Distinguish Thymus-Derived from Extrathymically-Induced CD4+Foxp3+ Regulatory T Cells. PloS One. 2015;10(10):e0141161 10.1371/journal.pone.0141161 26495986 10.1371/journal.pone.0141161 PMC4619666

[43] Akimova T, Beier UH, Wang L, Levine MH, Hancock WW. Helios expression is a marker of T cell activation and proliferation. PloS One. 2011;6(8):e24226 10.1371/journal.pone.0024226 21918685 10.1371/journal.pone.0024226 PMC3168881

[44] Himmel ME, MacDonald KG, Garcia RV, Steiner TS, Levings MK. Helios+ and Helios- cells coexist within the natural FOXP3+ T regulatory cell subset in humans. J Immunol Baltim Md 1950. 2013 3 1;190(5):2001–8. 10.4049/jimmunol.1201379 23359504

[45] Zizka J, Hrdý J, Lodinová-Zádníková R, Kocourková I, Novotná O, Sterzl I, et al Effect of breast milk of healthy and allergic mothers on in vitro stimulation of cord blood lymphocytes. Pediatr Allergy Immunol Off Publ Eur Soc Pediatr Allergy Immunol. 2007 9;18(6):486–94. 10.1111/j.1399-3038.2007.00563.x 17651385

[46] Hrdý J, Novotná O, Kocourková I, Prokešová L. Cytokine expression in the colostral cells of healthy and allergic mothers. Folia Microbiol (Praha). 2012 5;57(3):215–9. 22476868 10.1007/s12223-012-0112-y

[47] Súkeníková L, Černý V, Novotná O, Petrásková P, Boráková K, Kolářová L, et al Different capacity of in vitro generated myeloid dendritic cells of newborns of healthy and allergic mothers to respond to probiotic strain E. coli O83:K24:H31. Immunol Lett. 2017 9;189:82–9. 10.1016/j.imlet.2017.05.013 28554713 10.1016/j.imlet.2017.05.013

[48] Kim H-J, Barnitz RA, Kreslavsky T, Brown FD, Moffett H, Lemieux ME, et al Stable inhibitory activity of regulatory T cells requires the transcription factor Helios. Science. 2015 10 16;350(6258):334–9. 10.1126/science.aad0616 26472910 10.1126/science.aad0616 PMC4627635

[49] Delgoffe GM, Woo S-R, Turnis ME, Gravano DM, Guy C, Overacre AE, et al Stability and function of regulatory T cells is maintained by a neuropilin-1-semaphorin-4a axis. Nature. 2013 9 12;501(7466):252–6. 10.1038/nature12428 23913274 10.1038/nature12428 PMC3867145

[50] Battaglia A, Buzzonetti A, Monego G, Peri L, Ferrandina G, Fanfani F, et al Neuropilin-1 expression identifies a subset of regulatory T cells in human lymph nodes that is modulated by preoperative chemoradiation therapy in cervical cancer. Immunology. 2008 1;123(1):129–38. 10.1111/j.1365-2567.2007.02737.x 18028372 10.1111/j.1365-2567.2007.02737.x PMC2433274

[51] Chen L. Co-inhibitory molecules of the B7/CD28 family in the control of T-cell immunity. Nat Rev Immunol. 2004 5;4(5):336–47. 10.1038/nri1349 15122199 10.1038/nri1349

[52] Fife BT, Bluestone JA. Control of peripheral T-cell tolerance and autoimmunity via the CTLA-4 and PD-1 pathways. Immunol Rev. 2008 8;224:166–82. 10.1111/j.1600-065X.2008.00662.x 18759926 10.1111/j.1600-065X.2008.00662.x

[53] Watanabe N, Nakajima H. Coinhibitory molecules in autoimmune diseases. Clin Dev Immunol. 2012;2012:269756 10.1155/2012/269756 22997525 10.1155/2012/269756 PMC3446788

[54] McAlees JW, Lajoie S, Dienger K, Sproles AA, Richgels PK, Yang Y, et al Differential control of CD4 ^+^ T-cell subsets by the PD-1/PD-L1 axis in a mouse model of allergic asthma: Cellular immune response. Eur J Immunol. 2015 4;45(4):1019–29. 10.1002/eji.201444778 25630305 10.1002/eji.201444778 PMC4440042

[55] Kumar S, Verma AK, Das M, Dwivedi PD. A molecular insight of CTLA-4 in food allergy. Immunol Lett. 2013 1;149(1–2):101–9. 10.1016/j.imlet.2012.12.003 23254121 10.1016/j.imlet.2012.12.003

[56] Munthe-Kaas MC, Carlsen KH, Helms PJ, Gerritsen J, Whyte M, Feijen M, et al CTLA-4 polymorphisms in allergy and asthma and the TH1/ TH2 paradigm. J Allergy Clin Immunol. 2004 8;114(2):280–7. 10.1016/j.jaci.2004.03.050 15316504 10.1016/j.jaci.2004.03.050

[57] McGee HS, Yagita H, Shao Z, Agrawal DK. Programmed Death-1 antibody blocks therapeutic effects of T-regulatory cells in cockroach antigen-induced allergic asthma. Am J Respir Cell Mol Biol. 2010 10;43(4):432–42. 10.1165/rcmb.2009-0258OC 19901343 10.1165/rcmb.2009-0258OC PMC2951873

[58] Ito A, Kondo S, Tada K, Kitano S. Clinical Development of Immune Checkpoint Inhibitors. BioMed Res Int. 2015;2015:605478 10.1155/2015/605478 26161407 10.1155/2015/605478 PMC4486755

[59] Michot JM, Bigenwald C, Champiat S, Collins M, Carbonnel F, Postel-Vinay S, et al Immune-related adverse events with immune checkpoint blockade: a comprehensive review. Eur J Cancer Oxf Engl 1990. 2016 2;54:139–48. 10.1016/j.ejca.2015.11.016 26765102

[60] Ronchetti S, Ricci E, Petrillo MG, Cari L, Migliorati G, Nocentini G, et al Glucocorticoid-induced tumour necrosis factor receptor-related protein: a key marker of functional regulatory T cells. J Immunol Res. 2015;2015:171520 10.1155/2015/171520 25961057 10.1155/2015/171520 PMC4413981

[61] Nocentini G, Riccardi C. GITR: a modulator of immune response and inflammation. Adv Exp Med Biol. 2009;647:156–73. 10.1007/978-0-387-89520-8_11 19760073 10.1007/978-0-387-89520-8_11

[62] Palomares O, Martín-Fontecha M, Lauener R, Traidl-Hoffmann C, Cavkaytar O, Akdis M, et al Regulatory T cells and immune regulation of allergic diseases: roles of IL-10 and TGF-β. Genes Immun. 2014 12;15(8):511–20. 10.1038/gene.2014.45 25056447 10.1038/gene.2014.45

[63] Ray A, Khare A, Krishnamoorthy N, Qi Z, Ray P. Regulatory T cells in many flavors control asthma. Mucosal Immunol. 2010 5;3(3):216–29. 10.1038/mi.2010.4 20164832 10.1038/mi.2010.4 PMC3039023

[64] Smaldini PL, Orsini Delgado ML, Fossati CA, Docena GH. Orally-Induced Intestinal CD4+ CD25+ FoxP3+ Treg Controlled Undesired Responses towards Oral Antigens and Effectively Dampened Food Allergic Reactions. PloS One. 2015;10(10):e0141116 10.1371/journal.pone.0141116 26517875 10.1371/journal.pone.0141116 PMC4627767

[65] Edwards JP, Hand TW, Morais da Fonseca D, Glass DD, Belkaid Y, Shevach EM. The GARP/Latent TGF-β1 complex on Treg cells modulates the induction of peripherally derived Treg cells during oral tolerance. Eur J Immunol. 2016 6;46(6):1480–9. 10.1002/eji.201546204 27062243 10.1002/eji.201546204 PMC11022272

[66] Josefowicz SZ, Niec RE, Kim HY, Treuting P, Chinen T, Zheng Y, et al Extrathymically generated regulatory T cells control mucosal TH2 inflammation. Nature. 2012 2 8;482(7385):395–9. 10.1038/nature10772 22318520 10.1038/nature10772 PMC3485072

[67] Konkel JE, Chen W. Balancing acts: the role of TGF-β in the mucosal immune system. Trends Mol Med. 2011 11;17(11):668–76. 10.1016/j.molmed.2011.07.002 21890412 10.1016/j.molmed.2011.07.002 PMC3205325

[68] Konkel JE, Zhang D, Zanvit P, Chia C, Zangarle-Murray T, Jin W, et al Transforming Growth Factor-β Signaling in Regulatory T Cells Controls T Helper-17 Cells and Tissue-Specific Immune Responses. Immunity. 2017 4 18;46(4):660–74. 10.1016/j.immuni.2017.03.015 28423340 10.1016/j.immuni.2017.03.015 PMC12230991

[69] Zizka J, Hrdý J, Lodinová-Zádníková R, Kocourková I, Novotná O, Sterzl I, et al Effect of breast milk of healthy and allergic mothers on in vitro stimulation of cord blood lymphocytes. Pediatr Allergy Immunol Off Publ Eur Soc Pediatr Allergy Immunol. 2007 9;18(6):486–94. 10.1111/j.1399-3038.2007.00563.x 17651385

