# Supplementary figures and images for: Distinct characteristics of Tregs of newborns of healthy and allergic mothers
Source: PLoS One. 2018 Nov 26;13(11):e0207998. doi: 10.1371/journal.pone.0207998 (PMC6258229; doi:10.1371/journal.pone.0207998)

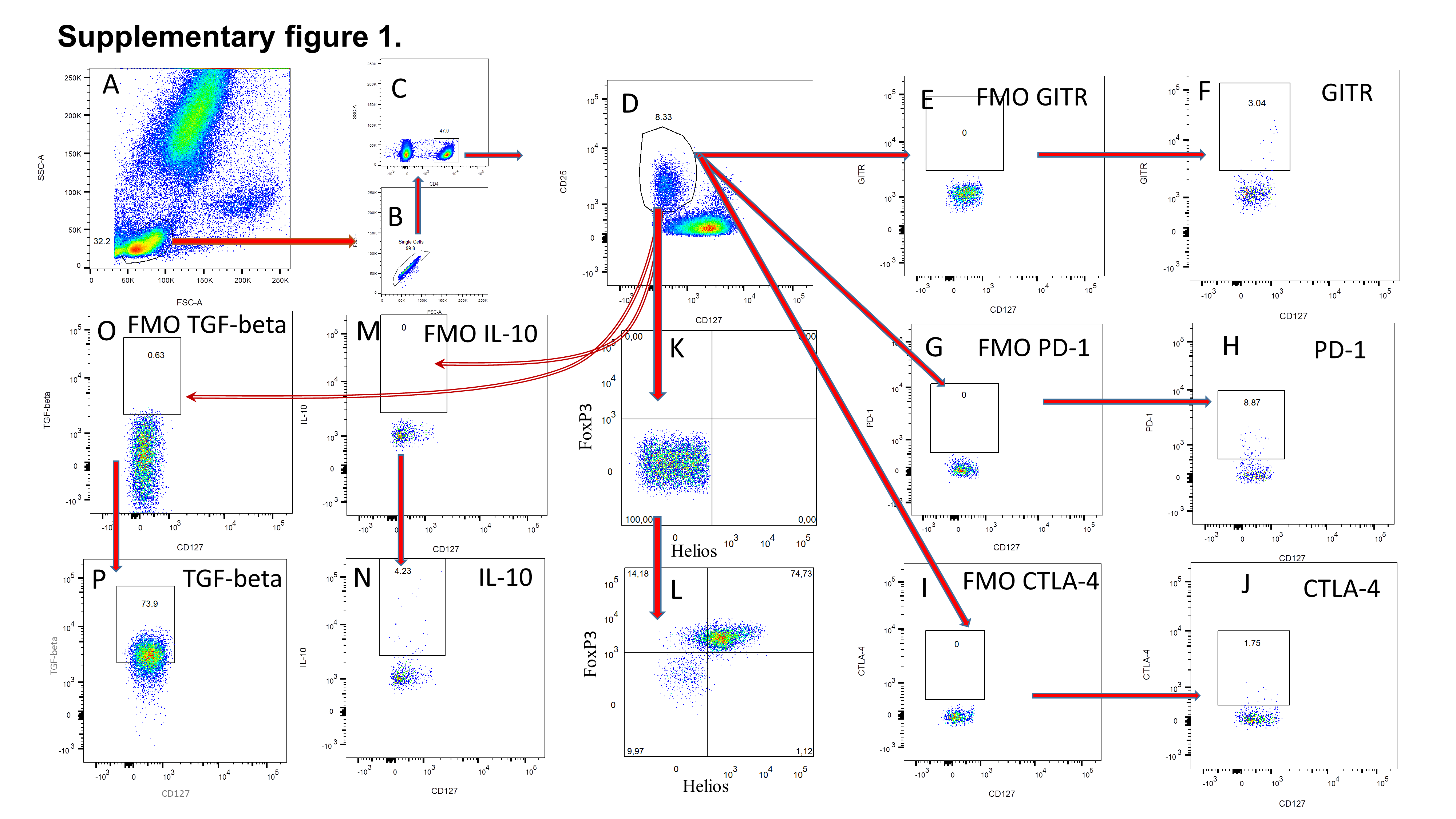

Supplement: S1 Fig — Cord blood samples were stained and analysed by flow cytometry. A-D: Gating strategy of CD4+CD25+CD127low cells.E-P: FMO controls and representative dot plots of CD4+CD25+CD127low cells stained for surface (E-J) and intracellular (K-P) markers. E, G, I: FMO controls for GITR, PD-1 and CTLA-4 staining. F, H, J: Representative dot plots of GITR, PD-1, CTLA-4. K: Control sample unstained for FoxP3 and Helios. L: Representative dot plot of intracellular staining for FoxP3 and Helios. M, O: FMO control for intracellular staining of IL-10 and TGF-β. N, P: Representative dot plot of intracellular staining for IL-10 and TGF-β. (TIF) [file pone.0207998.s001.tif]

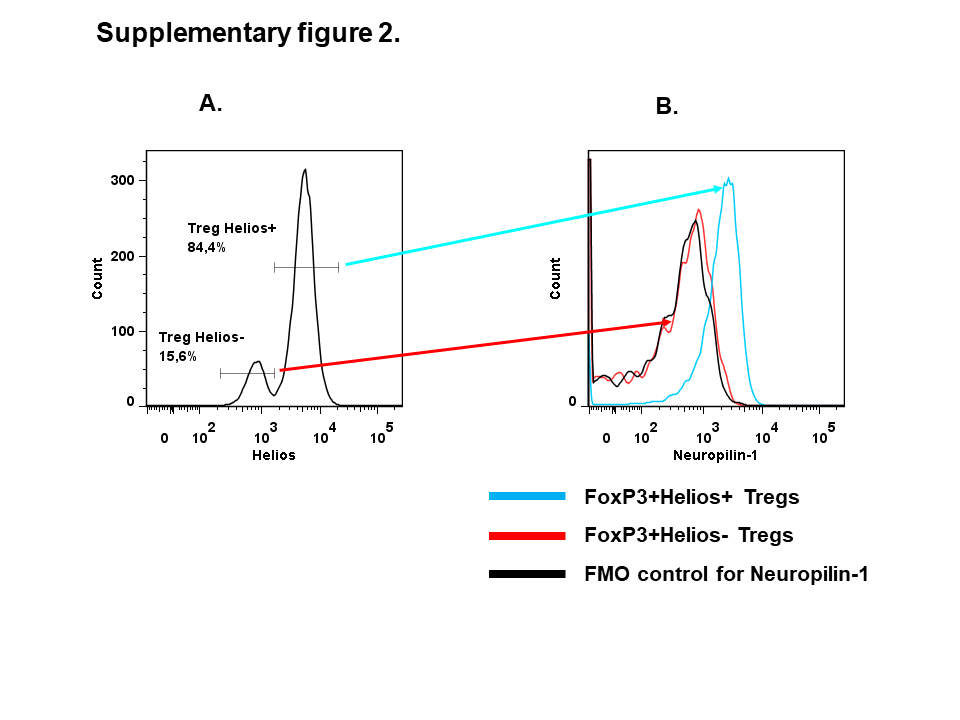

Supplement: S2 Fig — Several whole blood samples were stained for CD4, CD25, neuropilin-1, FoxP3 and Helios and analysed by flow cytometry. A: CD25highFoxP3+ cells were gated into Helios+ and Helios- populations. B: Expression of neuropilin-1 on Helios+ (blue) and Helios- (red) cells. FMO control for neuropilin-1 shown in black. (TIF) [file pone.0207998.s002.tif]

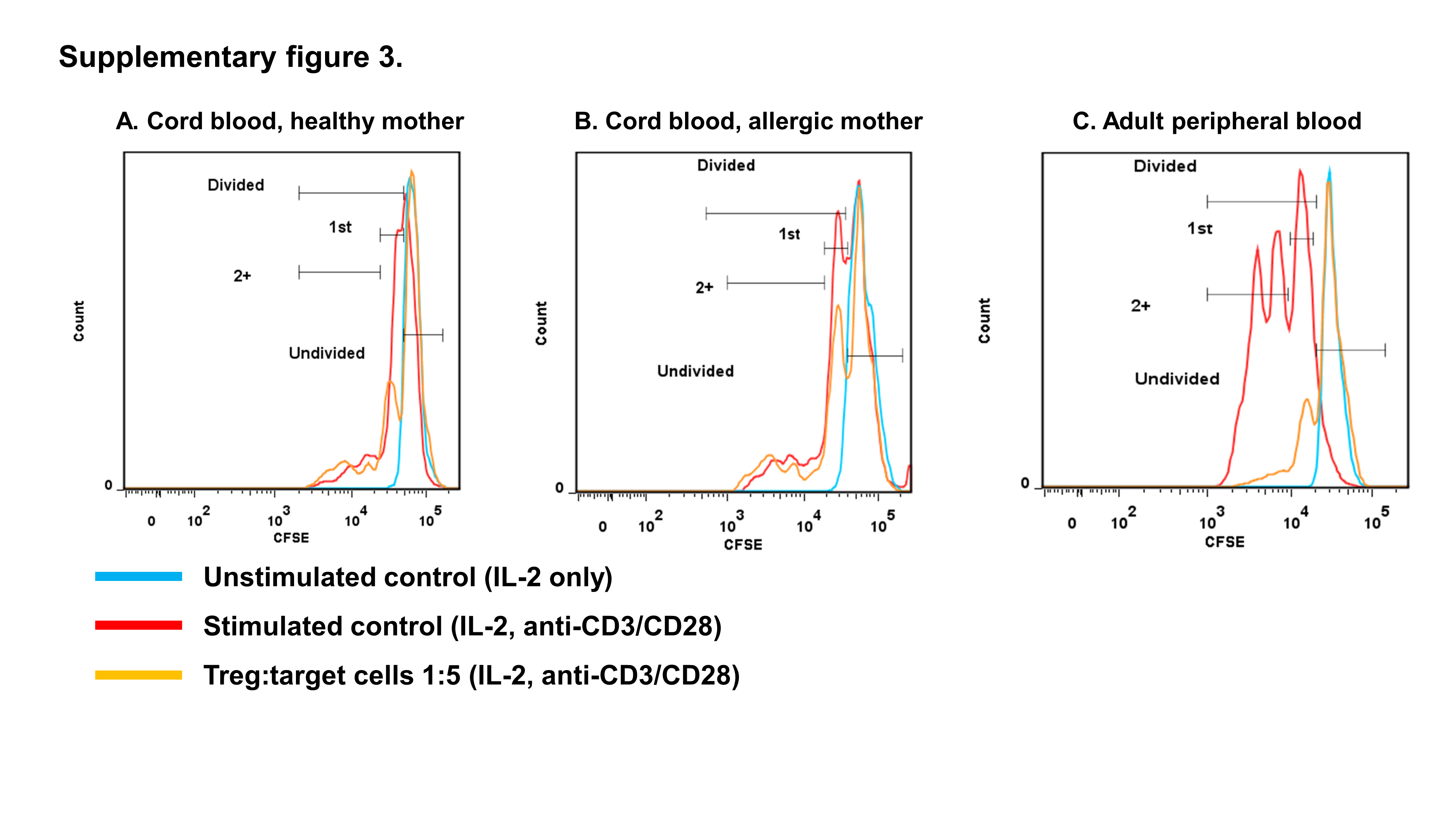

Supplement: S3 Fig — CD4+CD25-CD127+ target cells were magnetically isolated from cord blood mononuclear cells (n = 19), stained with 5 μM CFSE and cocultured with CD4+CD25+CD127low Treg cells at 1:5 Treg:target cell ratio. After 72 hours, cells were harvested, stained for CD4 and analysed by flow cytometry. Representative histograms show unstimulated control cells (blue), anti-CD3/CD28 stimulated control cells (red) and stimulated cells cocultured with Tregs at 1:5 Treg:target ratio (orange). A: Cells isolated from cord blood of a newborn of a healthy mother. B: Cells isolated from cord blood of a newborn of an allergic mother. C: Cells isolated from adult peripheral blood. (TIF) [file pone.0207998.s003.TIF]
