# Supplementary material for: Distinct characteristics of Tregs of newborns of healthy and allergic mothers
Source: PLoS One. 2018 Nov 26;13(11):e0207998. doi: 10.1371/journal.pone.0207998 (PMC6258229; doi:10.1371/journal.pone.0207998)
Supplement: S1 Table — CD4+CD25-CD127+ target cells were magnetically isolated from cord blood mononuclear cells (n = 19), stained with 5 μM CFSE and cocultured with CD4+CD25+CD127low Treg cells at 1:5 Treg:target cell ratio. After 72 hours, cells were harvested, stained for CD4 and analysed by flow cytometry. Table shows percentage of cells which went through at least one round of cell division (Divided cells), percentage of cells which did not proliferate (Undivided cells) and the number of peaks representing cell divisions in each sample (Number of generations). For each sample, allergy status is shown (A–children of allergic mothers, H–children of healthy mothers) and three conditions are included: Tregs cocultured with target cells at 1:5 Treg:target ratio; target cells stimulated with CD3 and CD28 monoclonal antibodies and IL-2; and unstimulated target cells, with only IL-2 added. (PDF) [file pone.0207998.s004.pdf]

Sample	Allergic status	Condition	Divided cells	Undivided cells	Number of generations
1	A	1:5 Treg:target	91.2	8.8	2
		Stimulated target cells	95.2	4.8	4
		Unstimulated target cells	89.5	10.5	1
2	H	1:5 Treg:target	69.2	30.8	3
		Stimulated target cells	81.0	19.0	4
		Unstimulated target cells	62.1	37.9	1
3	H	1:5 Treg:target	88.8	11.2	3
		Stimulated target cells	94.5	5.5	4
		Unstimulated target cells	88.6	11.4	1
4	H	1:5 Treg:target	86.5	13.5	2
		Stimulated target cells	94.0	6.0	2
		Unstimulated target cells	95.2	4.8	1
5	A	1:5 Treg:target	84.8	15.2	2
		Stimulated target cells	93.8	6.2	2
		Unstimulated target cells	83.6	16.4	1
6	A	1:5 Treg:target	96.8	3.2	3
		Stimulated target cells	99.2	0.8	5
		Unstimulated target cells	97.6	2.4	2
7	A	1:5 Treg:target	96.8	3.2	3
		Stimulated target cells	99.8	0.8	4
		Unstimulated target cells	77.6	22.4	2
8	A	1:5 Treg:target	47.0	53.0	4
		Stimulated target cells	51.5	48.5	4
		Unstimulated target cells	5.9	94.1	0
9	H	1:5 Treg:target	99.8	0.2	5
		Stimulated target cells	99.9	0.1	5
		Unstimulated target cells	93.7	6.3	2
10	H	1:5 Treg:target	99.3	0.7	5
		Stimulated target cells	99.8	0.2	5
		Unstimulated target cells	96.0	4.0	1
11	A	1:5 Treg:target	96.5	3.5	4
		Stimulated target cells	98.5	1.5	4
		Unstimulated target cells	90.0	10.0	1
12	A	1:5 Treg:target	91.5	8.5	4
		Stimulated target cells	94.3	5.7	4
		Unstimulated target cells	94.1	5.9	2
13	H	1:5 Treg:target	99.3	0.7	5
		Stimulated target cells	99.8	0.2	5
		Unstimulated target cells	96.0	4.0	1
14	H	1:5 Treg:target	38.9	61.1	4
		Stimulated target cells	51.7	48.3	4
		Unstimulated target cells	2.9	97.1	0
15	H	1:5 Treg:target	88.1	11.9	3
		Stimulated target cells	95.8	4.2	4
		Unstimulated target cells	13.1	86.9	1
16	H	1:5 Treg:target	84.6	15.4	6
		Stimulated target cells	90.6	9.4	6
		Unstimulated target cells	74.8	25.2	2
17	A	1:5 Treg:target	73.8	26.2	4
		Stimulated target cells	75.6	24.4	4
		Unstimulated target cells	63.0	37.0	1
18	H	1:5 Treg:target	65.8	34.2	3
		Stimulated target cells	71.7	28.3	4
		Unstimulated target cells	67.9	32.1	1
19	H	1:5 Treg:target	1.1	98.9	0
		Stimulated target cells	80.7	19.3	5
		Unstimulated target cells	67.4	32.6	4
